# Heart Rate Variability and Blood Pressure during Dynamic and Static Exercise at Similar Heart Rate Levels

**DOI:** 10.1371/journal.pone.0083690

**Published:** 2013-12-13

**Authors:** Matthias Weippert, Kristin Behrens, Annika Rieger, Regina Stoll, Steffi Kreuzfeld

**Affiliations:** 1 Institute of Preventive Medicine, University of Rostock Medical School, Rostock, Mecklenburg-Vorpommern, Germany; 2 Center for Life Science Automation, University of Rostock, Rostock, Mecklenburg-Vorpommern, Germany; University of Adelaide, Australia

## Abstract

Aim was to elucidate autonomic responses to dynamic and static (isometric) exercise of the lower limbs eliciting the same moderate heart rate (HR) response. Method: 23 males performed two kinds of voluntary exercise in a supine position at similar heart rates: static exercise (SE) of the lower limbs (static leg press) and dynamic exercise (DE) of the lower limbs (cycling). Subjective effort, systolic (SBP) and diastolic blood pressure (DBP), mean arterial pressure (MAP), rate pressure product (RPP) and the time between consecutive heart beats (RR-intervals) were measured. Time-domain (SDNN, RMSSD), frequency-domain (power in the low and high frequency band (LFP, HFP)) and geometric measures (SD1, SD2) as well as non-linear measures of regularity (approximate entropy (ApEn), sample entropy (SampEn) and correlation dimension D2) were calculated. Results: Although HR was similar during both exercise conditions (88±10 bpm), subjective effort, SBP, DBP, MAP and RPP were significantly enhanced during SE. HRV indicators representing overall variability (SDNN, SD 2) and vagal modulated variability (RMSSD, HFP, SD 1) were increased. LFP, thought to be modulated by both autonomic branches, tended to be higher during SE. ApEn and SampEn were decreased whereas D_2_ was enhanced during SE. It can be concluded that autonomic control processes during SE and DE were qualitatively different despite similar heart rate levels. The differences were reflected by blood pressure and HRV indices. HRV-measures indicated a stronger vagal cardiac activity during SE, while blood pressure response indicated a stronger sympathetic efferent activity to the vessels. The elevated vagal cardiac activity during SE might be a response mechanism, compensating a possible co-activation of sympathetic cardiac efferents, as HR and LF/HF was similar and LFP tended to be higher. However, this conclusion must be drawn cautiously as there is no HRV-marker reflecting “pure” sympathetic cardiac activity.

## Introduction

Three mechanisms are thought to be responsible for the neural cardiovascular modulation during voluntary muscle contractions: an activation of higher brain centers (“central command”) as well as reflex activity primarily involving inputs from chemo- and mechanoreceptor (“muscle metaboreflex”) and baroreceptor afferents (“baroreflex”) [1-9]. Each mechanism activates neuronal circuits within the medulla and thus modulates the sympathetic and parasympathetic outflow from the cardiovascular control center [1-8]. The influence of each mechanism on the heart rate and blood pressure response to exercise depends on factors like recruited muscle mass, muscle fiber type, exercise intensity and the exercise mode [10-12]. Early literature that compared the cardiovascular response to static and dynamic muscular actions indicated a strong increase in heart rate (HR) and systolic arterial pressure (SBP), and minor changes in diastolic arterial pressure (DBP) during dynamic work, while isometric work is thought to induce only modest increases of HR but marked increases in SBP and in particular DBP [13-18]. However, the generality of these observations is limited, because a) mostly cardiovascular effects of static (SE) and dynamic exercise (DE) had been studied separately; b) the quantification and thus the equating of workload during different exercise modes is difficult [18]; and c) often the compared muscles were of different size and location. Static vs. dynamic contractions of identical small muscles, e. g. during submaximal handgrip exercise, elicited similar HR and BP response [19], while it was shown that submaximal isometric contractions of larger muscles (e. g. knee extensors/flexors) at 40 % of maximum effort can induce lower HR and BP responses than isokinetic or isotonic contractions [20]. During moderate exercise intensity Chapman and Elliott found a significant increase in HR and SBP during DE, while DBP was highest during SE [18]. Nevertheless they concluded that when the same muscle groups are used the effect of the exercise modes on cardiovascular response is more similar than frequently stated. Gonzales-Camarena and colleagues compared DE (cycling) at 30 % VO_2_max and SE (isometric exercise of the knee extensors) at 30 % maximal voluntary contraction force (MCV). While respiratory rate was similar at the equivalent relative workloads they found different cardiovascular response pattern: a lower heart rate, a higher blood pressure response as well as higher effort perception for SE compared to DE [21]. In addition to the lower HR response also HRV measures pointed to a stronger vagal modulation during the SE [21]. Generally, compared to DE, isometric contractions might elicit a stronger chemoreflex response, as blood flow within and the release of metabolites from the muscle is limited. The chemoreflex elevates blood pressure by a sympathetic vasoconstriction [4], but also seems to affect sympathetic heart rate modulation [6].

To date, there are only few studies available that compared the cardiovascular response pattern at similar heart rates. Lindquist et al. found a stronger increase of SBP and DBP during isometric handgrip compared to cycling at comparable heart rates (about 90 bpm) [16]. Leicht and coworkers compared rating of perceived exertion (RPE) and cardio-respiratory response to dynamic muscular activity of different muscle groups at 50 % maximum HR (HR_max_) and 65 % HR_max_, respectively. The investigators found greater heart rate variability (HRV) and greater ratings of perceived exertion despite lower oxygen consumption during upper body dynamic exercise compared to lower or whole body dynamic exercise at similar heart rates [11]. They concluded that the greater HRV may represent greater vagal or dual autonomic modulation, but recommended further investigation of the underlying mechanisms.

Cottin et al. compared HRV indices during a judo randori vs. ergometer cycling eliciting the same heart rate level. They concluded that a) steady-state dynamic exercise or conversely exercise made of both isometric and irregular dynamic efforts can be distinguished by HRV analysis and b) based on their results of a similar spectral energy distribution within the LF and HF bands, “HR autonomic control during exercise depends on HR level rather than on exercise type“. Due to the study design in the Cottin et al. study including intense exercise at an average heart rate above 180 beats x min^-1^, conclusions regarding the autonomic mode of heart rate control based on spectral analyses of HRV are strongly limited. HRV at greater HR-levels is often almost negligibly and the remaining variance especially within the high frequency band is probably due to non-neural mechanisms [22-24]. Furthermore, the different location and size of the active muscles during cycling and judo exercises makes interpretation regarding the autonomic mode of heart rate control difficult.

In their case study of one world class dinghy sailor, Princi et al. found different autonomic modes of cardiac control based on HRV-analysis when comparing sailing and cycling at similar heart rates [25]. Because of higher values of the low frequency power (LFP) and the ratio of low to high frequency heart rate variance (LF/HF-ratio) in upwind sailing (static exercise) a different sympatho-vagal modulation of cardiac function – and therefore a different heart-brain interaction – under different exercise modes was suggested [25]. However, the generalizability of this statement is strongly limited, because only one athlete was subject of investigation and the muscle groups engaged were not similar during both exercises. 

Aim of this laboratory study was to assess the modes of autonomic control under DE vs. SE at similar, low heart rate level. HRV, the beat to beat fluctuation of heart rate, and blood pressure were used as non-invasive measures to elucidate the autonomic mechanism underlying cardiovascular control under the different experimental conditions [6,24,26,27]. Provided a different autonomic response pattern, we hypothesized that blood pressure and / or heart rate variability measures would be different during the different exercise modes. 

## Materials and Methods

### Ethics statement

Approval of the local ethics committee at the University of Rostock was obtained. 

### Participants

Twenty three healthy males were recruited by personal invitation and gave their informed written content to take part in this study. Table 1 shows selected characteristics of the participants. Aerobic fitness of the participants was estimated by the use of anthropometric and socio-demographic data, the self reported physical activity level as well as participant´s perceived functional ability [28]. All volunteers were physically active and healthy and none of them took medication. They abstained from any exhaustive exercise and alcohol for 24 h prior to the experiment. Further, the consumption of caffeine or nicotine was not allowed during the night and on the morning of the experiment [29]. 

**Table 1:** Characteristics of the participants (n = 23).

	**Age** [yrs]	**Weight** [kg]	**Height** [m]	**BMI** [kg/m^2^]	**Relative VO_2_max** [ml*min^-1^*kg^-1^]
**Mean**	**25.5**	**84.0**	**185.4**	**24.3**	**51.1**
(SD)	(2.6)	(7.7)	(5.9)	(1.5)	(3.5)
Range	21 – 32	72.0 – 100.0	1.74 – 1.95	22.3 – 27.2	43.7 – 58.3

### Protocol

**Figure 1:**
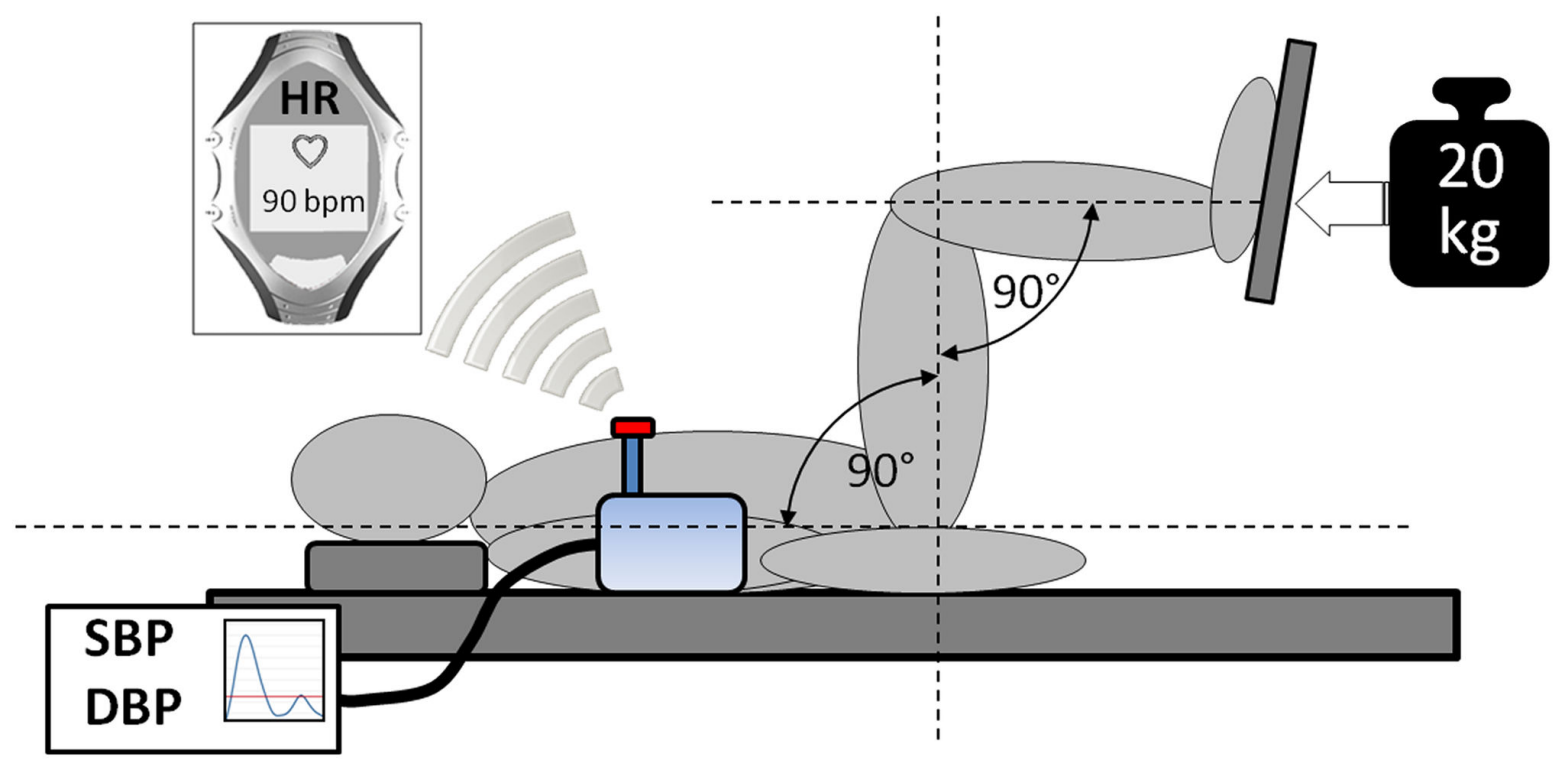
Scheme of the experimental setup during isometric leg exercise. Heart rate was monitored continuously using a wireless chest belt system (Polar® RS800). Blood pressure was measured discontinuously minute by minute.

**Figure 2:**
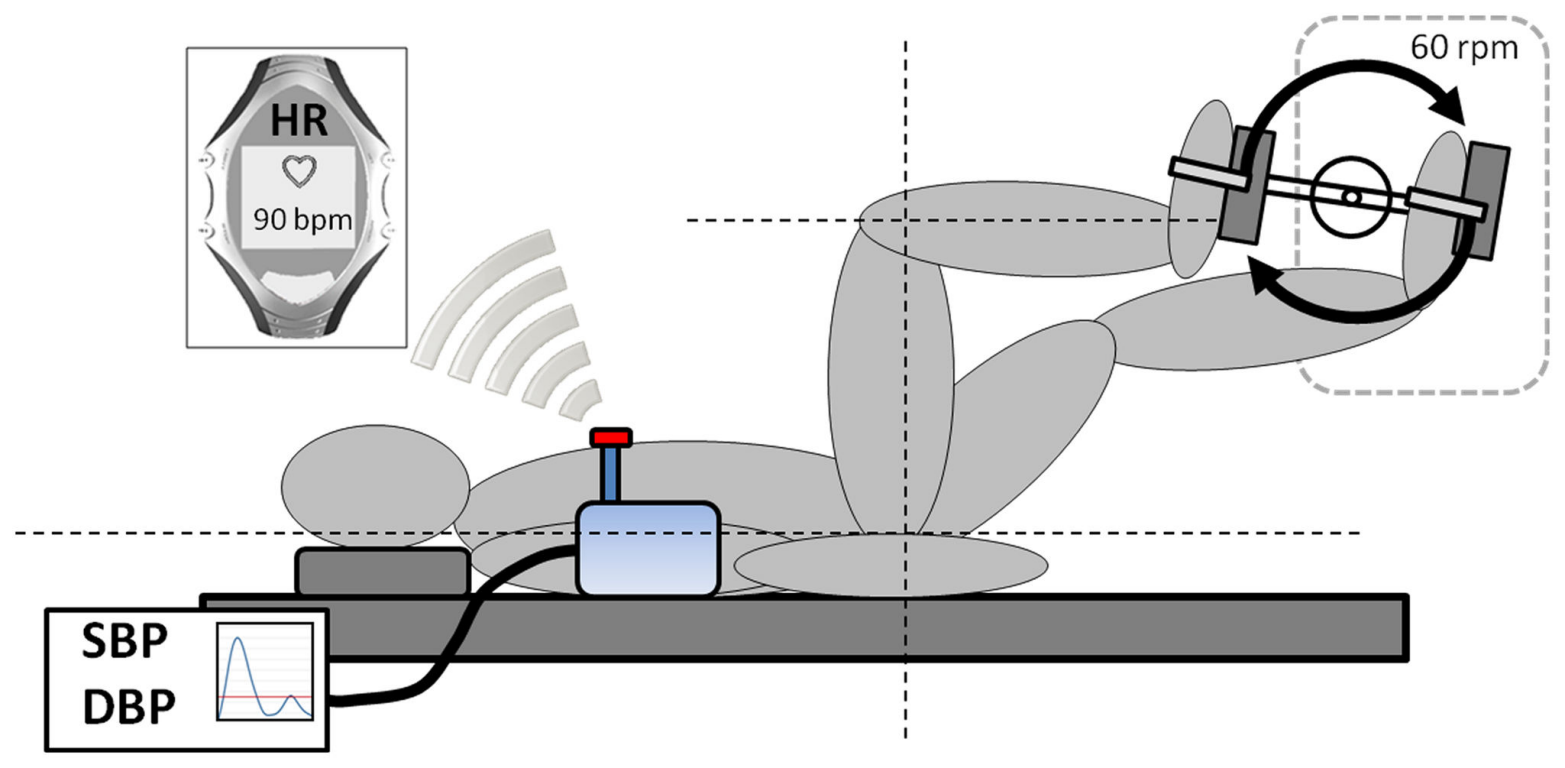
Scheme of the experimental setup during dynamic leg exercise using a cycle ergometer (Ergoline® ER 900). Pedaling revolutions were set at 60 rpm; resistance was individually adapted to match a heart rate level similar to the isometric exercise.

Participants underwent exercise testing on two occasions. Testing was carried out in supine position (Figure 1) at the same time of day and the same weekday to minimize confounding effects on the autonomic nervous system emerging from diurnal influences and daily activity patterns [30-32]. Each exercise session lasted five minutes and was carried out after a 5-minute adaptation period in a supine position to exclude orthostatic effects on autonomic regulatory control. All sessions included the measurement of SBP and DBP using the automatic blood pressure measuring device Bosotron 2, (boso Inc., Germany) [33]. Mean arterial pressure (MAP) was calculated by (SBP + 2*DBP)/3. Heartbeat intervals were measured using the RS800 heart rate monitor (Polar Inc., Finland), a chest belt system with an accuracy of 1 millisecond [34,35]. Isometric leg press had to be performed with a weight of 20 kg while knee flexion was 90° (Figure 1). This relatively low weight was chosen because preliminary testing indicated that this intensity could be maintained for five minutes without eliciting valsalva manoeuvres and interruptions to rest. Further, this low intensity resistance exercise allowed the adjustment of a physiological steady state within one minute. One week after the SE session DE was carried out. Cycling cadence during DE was held constant at 60 revolutions per minute and resistance of the ergometer was individually adjusted to match the individual heart rate response elicited by SE (Figure 2). Heart rate was monitored continuously by the investigator using the RS800 heart rate monitor. Average power output was 46.7 ± 19.5 Watts (range: 20 to 75 W). Immediately after each exercise session participants rated their individual physical effort on a scale from 0 (unexacting) to 10 (maximal exhaustion).

### Data processing

To ensure steady state conditions only the last three minutes of each session were analyzed. Blood pressure was measured minute-by-minute for the last 3 minutes of the exercise sessions and averaged for statistical analysis. Heart rate was measured beat-by-beat and averaged for 3 minutes as well. A short term HRV-analysis was performed for 3-minute RR-interval segments during steady-state conditions. 

### HRV measures

RR-interval series were processed using the free software Kubios HRV 2.0 (University of Kuopio, Finland). All analyzed RR-time series exhibited low noise (rate of erroneous RR-intervals below 5 %). Before the computation, RR-time series were corrected for artifacts using adaptive filtering and detrended (detrending method: smoothn priors, λ500). Time domain (SDNN, RMSSD) and Poincaré Plot indices (SD 1, SD 2) were calculated. Frequency domain analysis (LFP, HFP, and their normalized values LF n.u., HF n.u., LF/HF ratio) were performed using a Fast Fourier Transform (Welch’s periodogram: 256 s window with 50% overlap) [27]. Further, approximate entropy (ApEn), sample entropy (SampEn) and correlation dimension D_2_, nonlinear measures of regularity and complexity of physiological time series, were calculated [36-39]. ApEn is a measure of regularity of the RR-interval series with irregularity resulting in high and regularity in low values, respectively. It measures the likelihood that runs of patterns that are close to each other will remain close in the next incremental comparisons [40]. SampEn is similar to ApEn but less dependent on record length [41]. Its calculation relies on counts of *m*-long templates matching within a tolerance *r* that also match at the next point. For SampEn calculation the value of *m* was selected to be *m* = 2, for tolerance *r* a fraction of the standard deviation of the RR-data (*r* = 0.2*SDNN) was chosen [40]. Low values of SampEn arise from extremely regular time series, higher values reflect more complexity, and highest values are typical for stochastic data sets [42-44]. Correlation dimension D_2_ is expected to give information on the minimum number of dynamic variables needed to model the underlying system [45]. A lower value can be found, when the analyzed signal provides a higher regularity, higher values are supposed to reflect higher complexity or randomness [46]. Nonlinear parameters have proven their prognostic value in clinical settings [47-49] but the physiological background of their behavior is not very well established. Despite the fact, that the autonomic nervous system seems to be the main modulator [50,51], often there is no correlation with traditional HRV-indices [49,52,53]. 

T-test analysis for matched pairs was carried out to test for differences of the means (SPSS 15.0). Further, the effect size for significant differences between SE and DE were calculated using G*Power 3.1 (Düsseldorf University, Germany) [54].

## Results

Compared to DE, during SE subjective effort, blood pressure (SPB, DBP and MAP), RPP and the HRV indices SDNN, RMMSD, HFP, SD 1 and SD 2 were significantly enhanced (Table 2). Heart rate complexity measures ApEn and SampEn were significantly reduced, whereas D_2_ was significantly increased during SE (Table 2). RR-interval and thus, heart rate did not differ between SE and DE. The 95% CI of the paired heart rate differences ranged from -0.18 to 0.75 beats/min (p = 0.221). 

**Table 2:** T-test statistics and effect sizes for pair wise comparisons of heart rate, blood pressure, heart rate variability and subjective effort during static (SE) and dynamic exercise (DE).

**Parameter^*#*^**	**Static Exercise (SE)**	**Dynamic Exercise (DE)**	**T**	**df**	**p-value**	**Effect size**
Heart Rate [bpm]	88.0 ± 9.7	87.7 ± 9.5	1.261	22	0.221	0.261
**SBP [mmHg]**	**158.0 ± 12.1**	**140.2 ± 15.3**	**7.794**	22	**0.000**	**1.63**
**DBP [mmHg]**	**95.3 ± 6.3**	**68.5 ± 8.2**	**15.671**	22	**0.000**	**3.27**
**MAP [mmHg]**	**116.2 ± 6.1**	**92.4 ± 8.8**	**15.491**	22	**0.000**	**3.21**
**RPP [mmHg/min]**	**13932.1 ± 2100.8**	**12340.5 ± 2126.3**	**8.763**	22	**0.000**	**1.83**
RR-interval [ms]	691.8 ± 80.4	692.8 ± 77.9	-0.511	22	0.614	0.10
**SDNN [ms]**	**23.1 ± 6.6**	**16.7 ± 6.1**	**3.826**	22	**0.003**	**0.75**
**RMSSD [ms]**	**16.9 ± 8.5**	**13.1 ± 6.9**	**2.966**	22	**0.021**	**0.62**
**HFP [ms^2^]**	**142.8 ± 145.0**	**52.4 ± 45.0**	**3.080**	**22**	**0.005**	**0.65**
LFP [ms^2^]	414.1 ± 256.2	251.8 ± 339.5	1.921	22	0.068	0.40
HF n.u.	25.1 ± 16.5	20.6 ± 15.9	1.064	22	0.299	0.22
LF n.u.	74.9 ± 16.5	79.4 ± 15.9	-1.064	22	0.299	-0.22
LF/HF	5.8 ± 5.7	6.6 ± 4.7	-0.549	22	0.588	0.00
**SD 1**	**12.1** ± 6.0	**9.4** ± 4.9	**2.977**	**22**	**0.007**	**0.50**
**SD 2**	**42.9** ± 29.1	**29.1** ± 10.8	**4.770**	**22**	**0.000**	**0.99**
**ApEn**	**0.90** ± 0.14	**0.99** ± 0.10	**-3.501**	**22**	**0.002**	**-0.73**
**SampEn**	**1.18 ± 0.36**	**1.43 ± 0.31**	**-4.997**	**22**	**0.000**	**-1.04**
**D_2_**	**1.28 ± 1.06**	**0.44 ± 0.83**	**4.570**	**22**	**0.000**	**1.00**
**Subjective effort^*##*^**	**4.4 ± 1.6**	**2.3 ± 1.1**	**5.786**	**22**	**0.000**	**1.208**

^#^ Parameters showing significant differences between the means of the two exercise modes are indicated by bold letters

^##^ Effort was rated on a scale from 0 (= minimal) to 10 (= maximal)

## Discussion

Aim of this study was to elucidate the mechanisms of autonomic response during static and dynamic muscular work of the lower limb at the same heart rate level by analyzing blood pressure and heart rate variability. Despite eliciting the same heart rate response subjective effort, blood pressure and heart rate variability differed significantly between the two exercise modes. RPP – an indirect measure of myocardial oxygen consumption – was increased by 13% under SE [55,56]. Generally, findings of this study suggest that – provided a similar low heart rate level – autonomic control processes are qualitatively different during dynamic and static work of the same large muscles. 

Because heart rate was similar, the increased blood pressure during SE can principally be attributed to an increased peripheral vasoconstriction and/or changes in stroke volume. Especially metabolite accumulation in the isometric working muscle (muscle metaboreflex) but also changes in central command can lead to the enhanced blood pressure response observed under SE [4,57-65]. As the sense of effort and the perception of afferent sensory inputs appear to be closely related during most exercise – despite being based on different neurological mechanisms [66] – the stronger perception of effort during SE can support the conclusion of an increased metaboreceptor feedback during SE. On the other hand it cannot be excluded that a change of central command, mirrored by the increase in subjective effort, might have contributed to the elevation of blood pressure during SE [1,2,4,8,66-68]. However, the muscle metaboreflex has been suggested to be the dominant mechanism responsible for the vasculature response (blood pressure increase), whereas the central command is supposed to be the main modulator of the cardiac response (heart rate increase) during SE [4]. There are several human studies which showed stroke volume to be unchanged or even decreased during mild to moderate exercise during both exercise modes [62,63,69]. Thus, the change in vasomotor tone and not stroke volume seems to be the main modulator of the different blood pressure response observed during SE and DE. Most likely the muscle metaboreflex overrides the baroreflex, leading to a stronger sympathetic efferent activity to the vessels during SE [4,58,64,70-74].

While the blood pressure response seems to evidence increased sympathetic efferent drive to the vessels during SE, the vagally related HRV-measures RMSSD, HFP and SD 1 were significantly increased, speaking for an elevated vagal cardiac modulation. At the same time the LF/HF ratio – a measure thought to reflect sympathovagal balance [27] – and heart rate were unchanged. Also SDNN, SD 2 and LFP – all influenced by sympathetic and parasympathetic efferent activity – were higher or tended to be higher, respectively. These results partly suggest an increase in sympathetic heart rate modulation under SE as well. Other studies have supported the view of an increased dual autonomic modulation during SE compared to DE at similar relative work or heart rate. By studying heart rate variability during post-exercise ischemia (metaboreflex model), Nishiyasu et al. concluded that parasympathetic cardiac tone is enhanced, and thus HRV was increased, to balance enhanced sympathetic cardiac activity [75]. Also Gonzalez-Camarena and colleagues suggest an increased vagal outflow due to the baroreflex following a sympathetic activation [21]. Further, recent animal studies suggest, that even at the start of exercise physiological response is not exclusively elicited by vagal withdrawal, but also by increased sympathetic activity [7]. According to the idea of an autonomic space, a reciprocal behavior, e. g. vagal withdrawal and a concomitant sympathetic activation, is only one of many autonomic modes, potentially modifying heart rate during exercise. Sympathetic-parasympathetic co-activation, sole vagal withdrawal or sympathetic enhancement can have the same net-effect on heart rate as reciprocal relations of the autonomic branches as well [64,75-77]. Taken together the results of the HRV time and frequency domain analysis clearly indicated enhanced parasympathetic heart rate modulation during SE compared to DE. Although some observations indirectly support the assumption of an increased sympathetic cardiac activity, such conclusion must be drawn cautiously, as there is no HRV-marker reflecting “pure” sympathetic cardiac nerve traffic [78-81].

Complementary to traditional HRV-measures, which give information on the magnitude of the variability or important rhythms, non-linear indices are able to identify complex patterns of the analyzed time series. In our experiment the non-linear parameters differed significantly between the two exercise modes, according to the traditional vagal HRV-indices. D_2_ was significantly higher during SE, indicating higher complexity of the RR-time series compared to DE. Some researchers suppose that higher D_2_ values represent a stronger, interplay between the autonomic branches, e. g. in healthy vs. unhealthy cardiac states [37,82]. It can be speculated that an increased sympathetic-parasympathetic interaction and / or co-activation during SE, caused by afferent feedback from chemo- and baroreceptors, contributes to a more complex autonomic heart rate modulation. During DE heart rate might be modulated in a more reciprocal fashion leading to lower D_2_ values [76,77]. However, the decrease of ApEn and SampEn during SE seems to contradict the interpretation of the D_2_ values, as these results indicate higher complexity / lower regularity under DE. There is some evidence from other studies analyzing RR-data, showing only weak to medium or even no correlation between entropy measures and correlation dimension D_2_ [46,83]. Results of our experiment support the view that beyond their distinct mathematical calculation also the physiological background of the calculated regularity measures is different. The cause for ApEn and SampEn values being significantly higher during DE remains speculative. Mäkikallio et al. compared ApEn and other HRV indices of patients with a previous myocardial infarction and healthy controls and found enhanced ApEn in the patients. They suggested a more irregular breathing pattern as a possible cause for enhanced values of ApEn [53]. Pentillä and coworkers found that vagal blockade largely and slow breathing slightly decreases ApEn [50]. Further, during incremental dynamic exercise ApEn increased from the start to the end with and without parasympathetic blockade, indicating that non-vagal influences contribute to changes in ApEn [84,85]. Whether the significant differences of HRV complexity / regularity in our experiment are caused by different breathing patterns, changes in the sympathetic-parasympathetic interaction, or other causes remains to be investigated. Based on the finding of significant differences of the traditional vagally related HRV indices, a contribution of the cardiac vagal efferent activity to the distinct non-linear HRV characteristics during DE and SE is rather likely. 

### Limitations of this study

The lack of controlled breathing conditions can be seen as a limitation of this study. Although HF-Power and other vagally related HRV-measures might be confounded by different breathing patterns [86], some researchers question significant effects of breathing especially on RMSSD [87,88]. Nishiyasu and colleagues found similar effects on HRV during controlled vs. uncontrolled breathing when investigating the effects of the metaboreflex after static exercise [75]. To minimize effects of breathing pattern in our experiment, participants were instructed to breath as normal as possible during the exercises. Furthermore, all participants were trained, active male students and could sustain the load for 5 minutes without indicating exhaustion. Thus, the applied resistance of 20 kg can be considered as low to moderate at best [13,89,90]. This low weight should have prevented or at least limited the occurrence of valsalva manoeuvres or other irregular breathing patterns. 

Cycling per se might have affected heart rate variability by cardio-locomotor coupling; however, the contribution of cardio-locomotor coupling is thought to be rather small under low workload conditions [91,92].

## Conclusions

To our best knowledge this is the first study systematically investigating autonomic responses to isometric and dynamic work of the lower limbs at matched low heart rate levels. Results evidence distinctive patterns of autonomic response for each exercise mode. The results of the blood pressure and HRV analysis in time and frequency domain contribute to the assumptions that a) SE at low HR levels leads to a stronger vasoconstriction than DE and b) the vagal cardiac efferent activity is enhanced during SE. Whether the enhanced vagal activity balances the effect of an increased sympathetic drive to the vessels and / or to the heart remains to be elucidated. Although some observations indirectly support the assumption of an increased sympathetic cardiac activity, this conclusion must be drawn cautiously, as there is no HRV-marker reflecting “pure” sympathetic cardiac nerve traffic [27,78-81].
